# BioMEMS and Lab-on-a-Chip Course Education at West Virginia University

**DOI:** 10.3390/bios1010004

**Published:** 2011-01-20

**Authors:** Yuxin Liu

**Affiliations:** Lane Department of Computer Science and Electrical Engineering, Faculty member of WVNano Initiative, West Virginia University, 395 Evansdale Drive, Morgantown, WV 26506, USA; E-Mail: yuxin.liu@mail.wvu.edu; Tel.: +1-304-293-9144; Fax: +1-304-293-8602

**Keywords:** BioMEMS, Lab-on-a-Chip, microfluidics, graduate, undergraduate

## Abstract

With the rapid growth of Biological/Biomedical MicroElectroMechanical Systems (BioMEMS) and microfluidic-based lab-on-a-chip (LOC) technology to biological and biomedical research and applications, demands for educated and trained researchers and technicians in these fields are rapidly expanding. Universities are expected to develop educational plans to address these specialized needs in BioMEMS, microfluidic and LOC science and technology. A course entitled *BioMEMS and Lab-on-a-Chip* was taught recently at the senior undergraduate and graduate levels in the Department of Computer Science and Electrical Engineering at West Virginia University (WVU). The course focused on the basic principles and applications of BioMEMS and LOC technology to the areas of biomedicine, biology, and biotechnology. The course was well received and the enrolled students had diverse backgrounds in electrical engineering, material science, biology, mechanical engineering, and chemistry. Student feedback and a review of the course evaluations indicated that the course was effective in achieving its objectives. Student presentations at the end of the course were a highlight and a valuable experience for all involved. The course proved successful and will continue to be offered regularly. This paper provides an overview of the course as well as some development and future improvements.

## 1. Introduction

In recent years, the biological and biomedical applications of micro- and nanotechnology, such as Biological/Biomedical MicroElectroMechanical Systems (BioMEMS), microfluidics, and Lab-on-a-Chip (LOC) technology, have become increasingly prevalent and have found widespread use in a wide variety of applications such as diagnostics, therapeutics, and tissue engineering. The research and development activity in this field remains intense, and some applications have also been commercialized. [1] Accordingly, demands for educated and trained researchers and technicians in these fields are rapidly expanding, and universities are expected to develop educational plans and course curricula to address these specialized needs. At West Virginia University (WVU), biomedical research is performed within departments in the College of Engineering and Mineral Resources, the Health Science Center, and national labs, and centers around faculty collaborations. The students involved in this collaborative research can learn and obtain experience from specific assigned work. However, the number of students trained and involved in bio-related projects is limited, partially due to the limited number of research projects and amount of financial aid. Furthermore, the interdisciplinary nature of BioMEMS provides challenges from an educational perspective, which further limits student involvement in these emerging areas. BioMEMS, microfluidics, and LOC research and studies are highly interdisciplinary and require a significant background in biology, engineering, materials science, physics, chemistry, and mathematics. In addition, to provide students with an in-depth understanding of BioMEMS, labs and laboratory practices need to be specifically designed and developed together with the courses. 

**Figure 1:**
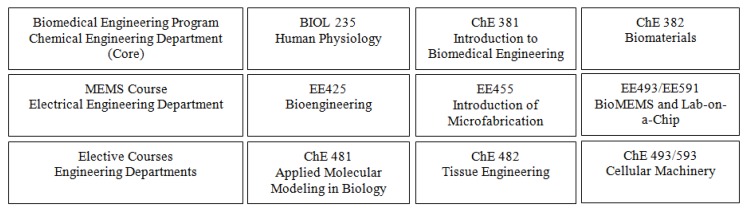
Biomedical engineering and MicroElectroMechanical Systems (MEMS) courses offered in departments of the College of Engineering and Mineral Resources at West Virginia University (WVU).

To satisfy the increasing requests for biomedical education from faculty and students at WVU, especially from undergraduate students, the College of Engineering and Mineral Resources is working to develop a series of courses in biomedical engineering, some of which have been taught for a few years. Figure 1 shows the titles and course numbers of these classes and their relationship to each other. Each of these courses in the sequence is focused on a topic area and provides significant depth in both theory and practice. Some courses include laboratory exercises. Some of these courses are designed for both undergraduate and graduate students and provide an in-depth education in biomedical engineering and related advanced technologies. 

To expose students to the emerging area of BioMEMS, a new course entitled *BioMEMS and Lab-on-a-Chip*, was recently developed in the Lane Department of Computer Science and Electrical Engineering at WVU. Due to the interdisciplinary nature and background requirements of this course, it was intended for graduate and advanced undergraduate students. Similar to courses offered at many other universities [2,3], the course focuses on the fundamental principles and applications of BioMEMS and microfluidic technologies in biology, medicine, and engineering, and provides a deeper coverage of the most advanced BioMEMS and LOC topics, such as BioMEMS materials, conventional MEMS fabrication and soft lithography, biosensors and biochips, microarray technology, microfluidics and its applications in cellular and tissue engineering, drug delivery and implant devices, and bio-nanotechnologies. In the first course offering, the enrollment expanded beyond the initial target audience of electrical engineering. The enrolled students had diverse backgrounds in electrical engineering, material science, biology, mechanical engineering, and chemistry. We effectively selected the most recently published review articles and research reports as supplemental materials, to provide students with a real-world perspective and to help them establish a keen sense of the most advanced developments in the field. Short quizzes at the beginning of lectures were used to test their understanding of key concepts and critical vocabulary. Each student, advised by the instructor, chose a research topic during the early part of the course and presented it at the end. The skills required for scientifically presenting research topics, such as literature searching, research article comprehension, PowerPoint presentation, and research paper writing (required for graduates), were also introduced to the students. Additionally, four lab sessions were arranged for instruction on more practical matters and fabrication details that were often glossed over in the lectures. We envisioned that the course would provide a strong fundamental base in biomedical microdevices and microsystems to the students who participated in the class.

## 2. Course Description

The course was designed as a dual-level course intended for both senior undergraduate and graduate students. To facilitate the enrollment of students from diverse backgrounds, there were no prerequisites other than senior class standing. Fourteen students were enrolled in the class, including two undergraduate and 12 graduate students, with backgrounds in electrical engineering, material science, biology, mechanical engineering, and chemistry. Although no experience with microfabrication, working in a Cleanroom, biology, or any other specialized areas were required, the diverse class made up of students with different backgrounds presented some instructional challenges. For example, there were essentially three groups of students. The students that already had microfabrication and Cleanroom experience expected to learn more about other aspects, such as how cells and macromolecules integrate within the devices from a biological and biomedical perspective. Other students who had a strong biology background preferred to learn how to use micro-scaled engineering approaches to solve biological problems and to understand the current limitations of conventional approaches. The third group of students did not have any of the microfabrication or biological experience of the other two groups, and expected the class to introduce them to some of the fundamental principles and applications from both perspectives. After considering these expectations and requirements, the class was initially introduced to important fundamental concepts and principles from both the micro-scaled engineering and biological perspectives. Further, the instructor emphasized correlations among these concepts and principles, helping the students to gain a general idea and understanding of these connections. The topics and their sequence are listed in Table 1. 

**Table 1:** Topics covered in the Biological/Biomedical MicroElectroMechanical Systems (BioMEMS) and lab-on-a-chip (LOC) course.

Week	Topics
1	Introduction and overview of BioMEMS and LOC
2	MEMS fabrication
3	BioMEMS fabrication and soft lithography
4	Lab: Cleanroom and lithography
5	Biological basics: cell, DNA, protein, biolinker
6	Biosensor and biochips
7	Review and Middle Exam
8	Microfluidics and components
9	Lab: microfluidic device fabrication and testing
10	Drug delivery and implant devices
11	Microtechnology and cells
12	Microtechnology and tissue
13	Bionanotechnology
14	Student oral presentations
15	Student oral presentations
16	Review and Final Exam

The sequence of topics was selected with three criteria in mind after referring to similar courses offered at other universities [2,3]. The first topic was an introduction to the fundamentals of MEMS and BioMEMS fabrication. Thus, the first three weeks of the course focused on introducing key concepts, such as conventional MEMS materials, various lithography methods, and bulk and surface micromachining. Then, lectures on nonconventional fabrication technologies (soft lithography and hot embossing) with a focus on biomedical applications were presented. After all necessary microfabrication technologies were introduced, the students were arranged for two lab sessions (I and II) in a class-100 and -1000 Cleanroom for a tour and a photolithography demonstration. The students were divided into two groups. During the lab session I, the Cleanroom manager toured the students through the Cleanroom and introduced different manufacturing equipments, including mask aligner, sputtering station, inductively coupled plasma (ICP) dry etcher, *etc*. In the lab session II, the technician introduced and demonstrated the standard photolithography process, including photoresist coating and baking, UV exposure, patterns development, *etc*. After three weeks, students were familiar with some fundamental structures and techniques. The theories and concepts were revisited later in the course when discussing any related devices, such as stress cantilever biosensors and their biomedical applications. After introducing microfluidic theories, important parameters, and components (such as microvalves and micropumps), another two lab sessions (III and IV) were arranged to demonstrate the fabrication and testing of microfluidic devices. In lab session III, the instructor demonstrated and taught the students the process of soft lithography, including polydimethylsiloxane (PDMS) mixing, degassing, pouring onto the mold, baking, punching holes for tubing connections, and bonding the PDMS device to a glass slide after oxygen plasma treatment. The microfluidic devices fabricated in lab session III were tested during lab session IV, and students were able to manipulate the different fluids speeds and observe how fluids flow and mix in channels. We found that the practice in labs helped to emphasize the concepts taught in the course, such as the laminar flow. The microfabrication flow chart and the experimental set up for microfluidic laminar flow testing were provided in the supplementary materials. The discussion of MEMS and BioMEMS fabrication, and, in particular, the lab sessions, provided the students with a common starting point for the later introduction of biological and biomedical micro-scaled systems and devices.

The second criterion was to not overwhelm the engineering students by the breadth of the interdisciplinary topics encompassed in BioMEMS and microfluidics. Although some students were involved in research projects and exhibited an interest in biotechnology, the students had few courses outside their own departments. Thus, the fundamental knowledge and important concepts commonly used in molecular and cellular biology were introduced earlier in the course, such as the central dogma of molecular biology (transcription and translation), DNA hybridization and polymerase chain reaction (PCR) techniques, and biolinkers using DNA and proteins. In addition, in light of the fact that the majority of the students had engineering backgrounds, the sequence of lectures moved from more engineering-intense toward more biologically-intense instruction in BioMEMS and LOC throughout the course. Based on this criterion, for example, micro-scaled biosensors for molecular detection were discussed earlier than the more biology-intense cellular biosensors and tissue bioreactors, which were introduced later in the class.

The third and final criterion was to give students equal exposure to both theoretical principles and their biological and biomedical applications. For example, after introducing the fundamentals of microfluidics, their applications were immediately discussed. This theme of “from theory to application” was carried out throughout the course.

Homework, quizzes, and two exams were given and included a wide variety of question types designed to test the students’ understanding of the basic principles of the various BioMEMS fabrication methods and applications discussed in class. Selected sample questions from the quizzes and exams can be found in the Supplementary material. Overall, the instructor felt that the course materials were well understood, and some students were prepared to design new BioMEMS and microfludic components to integrate with current microsystems in their research projects.

Each student was required to orally present a research topic related to BioMEMS, microfluidics and LOC. The instructor selected several topics for students to choose from. The instructor also gave the students flexibility to choose topics beyond the provided list. Individual meetings were arranged to advise students and help them to select their topic. The topics presented by each student at the end of the course are listed in Table 2. The skills for scientifically presenting research topics were also introduced at the beginning of the class. The project topics covered the full range of the course lectures and involved the most advanced and popular topics in BioMEMS and LOC.

**Table 2:** Topics covered by the student oral presentations.

Number	Topics
1	Optofluidic photonic crystal biosensors
2	Surface acoustic wave devices for microfluidic transport and biosensor applications
3	Diagnosis of cervical cancer using microchips
4	Microfluidic chips for optical detection of biotargets using quantum dots
5	Retinal prosthesis devices as treatment for vision loss
6	Integration of adaptive neuromorphics with machine olfaction in medicine
7	An overview of machine olfaction for medical diagnosis
8	The basic understanding microfluidic components to integrate with linear biomolecular motor systems
9	Colloidal semiconductor nanocrystals as förster resonance energy transfer donors for biosensing
10	Microfluidic integration of GaN based LEDs
11	Monitoring of cellular metabolic activity on a chip
12	Blood cell analysis system
13	Microtechnology and nanomaterials toxicity
14	Scaffolding for neural tissue regeneration

## 3. Textbook and Additional Bibliography

The list of potential textbooks for a class in biomedical applications using BioMEMS, microfluidics and LOC is currently relatively short. In addition, the textbook authors emphasized different topics, in part because of their different expertise and backgrounds. Some topics of the books are more related to micro-scaled engineering principles and approaches, whereas others are related more to specific applications, such as in molecular biology and chemistry. In light of these reasons and the rapid development of BioMEMS and LOC in some emerging research areas, we did not choose a single book as the course textbook. Instead, we placed two books on reserve in the library for supplemental reading [4,5]. We distributed handouts and assigned the most recent published review articles and research papers for each topic covered in class (the list for additional bibliography can be found in the Supplementary material). All class reading assignments were posted online before each lecture. There was an average of two review/research papers available on each selected topic for further reading. The instructor’s teaching media included a combination of PowerPoint slides and a whiteboard, which permitted many colorful schematic diagrams, illustrations, and videos, offering multiple representations of the same material. Pedagogical research suggests that using multiple representations is more conducive to teach an audience with diverse backgrounds, as students tend to work best with their preferred representation [6].

## 3. Results and Discussion

The main objectives of the course were to have the students gain background knowledge of BioMEMS and microfluidic techniques for understanding the fabrication and function of micro-scaled biosystems, to become familiar with miniaturization concepts, to be able to research the state-of-the-art in selected BioMEMS topics, to present key results in a seminar, to formulate open questions, and to define a research direction or application of a particular technology. In addition to these normal educational objectives, the course aimed to rapidly prepare and train students at WVU to perform basic research in the area of BioMEMS, LOC, and microfluidics. The course evaluation and feedback was very positive, as shown in the Supplementary materials. Both the undergraduate and graduate students, and the students’ advisors, had a favorable impression of the course. They appreciated the effort put into providing an opportunity to observe cutting-edge research in the classroom.

The course materials, including lecture presentation handouts, papers, and book references, were carefully selected to include some functional applications and commercial products. This preparation was critical for a good understanding of the areas covered by the course. Student feedback showed that the examples that illustrated the principles, concepts, and technical methods were valuable in understanding the material and as preparation for homework questions, exams, and quizzes. However, due to the broad coverage of so many new topics in a short period of time and the multidisciplinary nature of the course, many students were discouraged by the fact that some topics were not discussed in depth. Complete mastery of BioMEMS and microfluidics is not reasonable due to the fast pace of this course. Ideally, students would take other introductory courses in biology and microfabrication to help them to understand the fundamental concepts. However, such prerequisites would limit access to the class for some students. 

In addition, student feedback revealed that the students had diverging opinions of the breadth and depth of the course through reading research articles assigned to supplement lectures. Graduate students exhibited a more thorough understanding of the material than undergraduates, which was based on a more effective reading of the assignments. However, this process was a new experience for undergraduate students. In future course offerings, discussion of selected papers will be arranged to help undergraduate students understand the material better and improve their scientific reading skills. 

Standard WVU student evaluations of the course revealed high levels of support for the course and the instructor. In all aspects, the course was rated above average in the College and the University. Selected evaluations related to the course study are provided in the Supplementary materials. The course evaluation also revealed that many students were enthusiastic about lab sessions where they could observe the construction and testing of devices. Most students wanted to be able to do some of the experiments themselves and carry out some of the fabrication procedures shown during the labs. Currently, only four lab sessions are associated with the course, and technicians and instructors demonstrate the procedures and give the students tours through the labs. Additional lab components might allow the students to gain significant laboratory practice and experience, which will be invaluable to both students and their research advisors. However, adding additional lab components require further funding support, lab space, and skilled lab assistants. We are considering applying for a grant from the program of Education Research and Curriculum Development of the National Science Foundation to develop a practical laboratory course for microfluidic and BioMEMS to enhance students’ hands-on experimental abilities, especially for undergraduates. 

The oral presentations given at the end of the course were the highlight of the class and received praise from the students. The presentations were the culmination of the students’ projects after three months of searching and reading papers, and allowed them the opportunity to publically present and explain their work. Each presentation was judged by the instructor and also by the students, based on a presentation rubric (see Supplementary materials). The rubric was distributed earlier as a guideline for preparing their presentations. Attending the presentation session and judging the other presentations were mandatory. All student presentations were put online with other course materials so that everyone can access them for future reference. The students significantly benefited from receiving feedback from the instructor and other students in the class.

Most importantly, in reviewing the topics of the student oral presentations, the main objective of the course seemed to have been met. Fourteen students took the class, including 12 graduate students and two undergraduate students. All of them chose the presentation topics in the area of BioMEMS, microfluidics, or bionanotechnology. One undergraduate student in the class enrolled in the WVU graduate program and is currently working on a thesis topic in biosensors using MEMS and microfluidics. Six graduate students consulted the instructor about the materials, fabrication, and experimental setup discussed in the class for BioMEMS and microfluidics. They considered the course is a valuable resource for their current and future research. 

## 4. Conclusions

In summary, the *BioMEMS and Lab-on-a-Chip* course successfully integrated research topics into the class lectures, developed appropriate background to introduce the students to the principles of microfluidics, microfabrication, and basic biology, and orientated the students to facilitate the application of BioMEMS and microfluidics techniques to their own research. Some students consulted the instructor to get help designing and fabricating microfluidic devices to solve their experimental problems after the class. The students’ enthusiasm about BioMEMS and microfluicics was critical to the success of the course, and their responses to the course were highly favorable. However, there is still significant room for improvement, including efforts to develop further in-depth coverage of the basic BioMEMS and LOC components, the creation of a hands-on experimental laboratory, and assistance in designing projects under advice of the instructor. The course will continue to be offered, and students with diverse background are always welcome. In addition, any recommendations or inquires are welcome.
